# Neural Responses to Central and Peripheral Objects in the Lateral Occipital Cortex

**DOI:** 10.3389/fnhum.2016.00054

**Published:** 2016-02-19

**Authors:** Bin Wang, Jiayue Guo, Tianyi Yan, Seiichiro Ohno, Susumu Kanazawa, Qiang Huang, Jinglong Wu

**Affiliations:** ^1^College of Computer Science and Technology, Taiyuan University of TechnologyTaiyuan, China; ^2^Graduate School of Natural Science and Technology, Okayama UniversityOkayama, Japan; ^3^School of Life Science, Beijing Institute of TechnologyBeijing, China; ^4^Key Laboratory of Convergence Medical Engineering System and Healthcare Technology, The Ministry of Industry and Information Technology, Beijing Institute of TechnologyBeijing, China; ^5^Department of Radiology, Okayama University Hospital, Okayama UniversityOkayama, Japan; ^6^Graduate School of Medicine, Dentistry, Pharmaceutical Sciences, Okayama UniversityOkayama, Japan; ^7^Key Laboratory of Biomimetic Robots and Systems, Ministry of Education, Beijing Institute of TechnologyBeijing, China

**Keywords:** lateral occipital cortex, retinotopic maps, object category, fMRI, wide-view visual field

## Abstract

Human object recognition and classification depend on the retinal location where the object is presented and decrease as eccentricity increases. The lateral occipital complex (LOC) is thought to be preferentially involved in the processing of objects, and its neural responses exhibit category biases to objects presented in the central visual field. However, the nature of LOC neural responses to central and peripheral objects remains largely unclear. In the present study, we used functional magnetic resonance imaging (fMRI) and a wide-view presentation system to investigate neural responses to four categories of objects (faces, houses, animals, and cars) in the primary visual cortex (V1) and the lateral visual cortex, including the LOC and the retinotopic areas LO-1 and LO-2. In these regions, the neural responses to objects decreased as the distance between the location of presentation and center fixation increased, which is consistent with the diminished perceptual ability that was found for peripherally presented images. The LOC and LO-2 exhibited significantly positive neural responses to all eccentricities (0–55°), but LO-1 exhibited significantly positive responses only to central eccentricities (0–22°). By measuring the ratio relative to V1 (RRV1), we further demonstrated that eccentricity, category and the interaction between them significantly affected neural processing in these regions. LOC, LO-1, and LO-2 exhibited larger RRV1s when stimuli were presented at an eccentricity of 0° compared to when they were presented at the greater eccentricities. In LOC and LO-2, the RRV1s for images of faces, animals and cars showed an increasing trend when the images were presented at eccentricities of 11 to 33°. However, the RRV1s for houses showed a decreasing trend in LO-1 and no difference in the LOC and LO-2. We hypothesize, that when houses and the images in the other categories were presented in the peripheral visual field, they were processed via different strategies in the lateral visual cortex.

## Introduction

Humans have the ability to recognize objects quickly and efficiently over a large proportion of the visual field without needing to make eye movements. This object recognition ability decreases robustly with increasing eccentricity or viewing angle (Larson and Loschky, 2009; Strasburger et al., 2011; Yao et al., 2011; Yoo and Chong, 2012). Object recognition is thought to be mediated by hierarchical processing in the visual cortex (V1), where signals pass from the primary V1 to the ventral and lateral visual cortices (Grill-Spector, 2003; Schwarzlose et al., 2008). Functional magnetic resonance imaging (fMRI) studies have characterized multiple regions in the ventral and lateral visual cortices according to their consistent preferential responses to object categories. These regions include the lateral occipital complex (LOC), which preferentially responds to object vs. nonobject images (Grill-Spector et al., 1999; Riesenhuber and Poggio, 1999; Grill-Spector, 2003; Sayres and Grill-Spector, 2008), face-selective areas (fusiform face area, FFA; Kanwisher et al., 1997), and house-selective areas (parahippocampal place area, PPA; Epstein and Kanwisher, 1998). Investigations of the functions of these category-selective areas have contributed to our understanding of the neural mechanisms of object perception (Hasson et al., 2002; Grill-Spector, 2003; Schwarzlose et al., 2008; Wang et al., 2013b; Wu et al., 2013).

The LOC is located along the lateral occipital and temporal cortices, which exhibit retinotopic representations. Using fMRI and a checker board retinotopic mapping stimulus, Larsson and Heeger (2006) identified two hemifield representation areas in the vicinity of the LOC, which they designated LO-1 and LO-2. The two areas lie anterior to dorsal V3 and posterior to the middle complex (MT+). LO-1 and LO-2 show clear polar angle and eccentricity representations. LO-2 exhibits a sudden transition from central to peripheral locations (Larsson and Heeger, 2006; Amano et al., 2009), and more recently, Sayres and Grill-Spector (2008) demonstrated that the LOC extends beyond the boundaries of the visual field maps of LO-1 and LO-2.

Behavioral analyses have indicated that visual working-memory performance for faces decreases from the central to the peripheral visual field, whereas the corresponding performance for buildings remains unchanged across different eccentricities of up to 40° when the images were presented on a wide-view field (Yoo and Chong, 2012). Eccentricity biases were also demonstrated in FFA and PPA: the FFA preferred stimuli located in the central visual field, whereas the PPA preferred stimuli located in the peripheral visual field (Levy et al., 2001; Hasson et al., 2002). In a previous study in which a wide-view presentation field was utilized, we identified decreased neural activation in both the FFA and PPA as stimulus eccentricity increased. The FFA exhibited a higher ratio relative to the V1 response (RRV1) than the PPA. Furthermore, the difference increased from the central to the peripheral visual field (Wang et al., 2013b), suggesting that neural activations to stimuli presented in wide-view fields differ from those to central visual stimuli. An fMRI study involving the presentation of stimuli in the central visual field demonstrated a category bias of the neural responses to objects (Sayres and Grill-Spector, 2008): animate categories (body parts, animals, and faces) elicited slightly higher neural responses than those evoked by inanimate categories (cars, sculptures, and houses). Moreover, analyses based on the mean response and on the voxel-wise patterns of the response in the LOC identified differences in the responses to different categories (Schwarzlose et al., 2008). These studies suggest that neural responses in later V1 exhibit selectivity to object categories. However, category biases in neural activations to objects in the peripheral visual field are not well understood.

**Figure 1:**
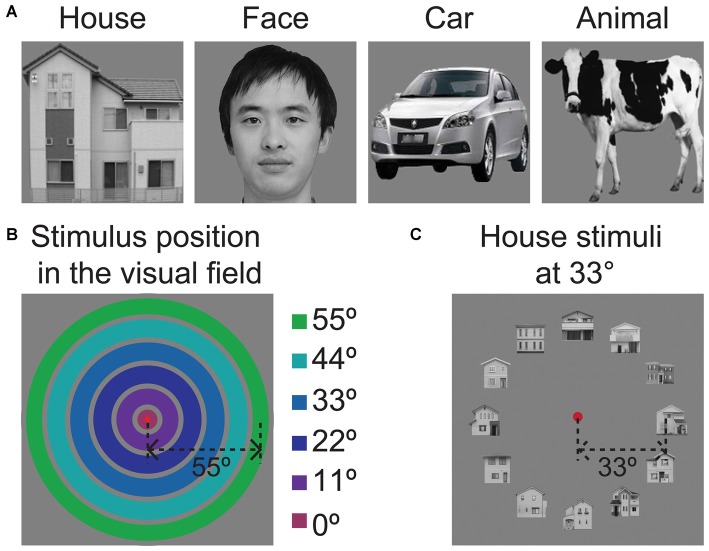
**Sample stimulus images used in the experiment. (A)** Sample image for each of the four object categories. The image of the face shown here does not depict the actual stimulus and is only intended to be an example. We have received written permission to use the photograph to illustrate the stimuli in publications. **(B)** Illustration of the six eccentricity positions of the object ring. The colored rings indicate the position of the object ring in the visual field. The degrees of eccentricity from the center fixation point are listed on the left side. **(C)** The presentation of images within the visual field, showing a ring of houses at an eccentricity of 33°.

In the present study, we used fMRI and a wide-view presentation system (Wang et al., 2013a; Wu et al., 2013) to study neural activations to central and peripheral objects in the lateral V1. During the MRI scanning, the subject was asked to view stimuli from four object categories (faces, houses, animals, and cars) that were arrayed in rings at six eccentricity levels within a visual field with 60° of eccentricity (Figure 1). The subjects were asked to categorize the images while maintaining fixation. We investigated the neural activation maps and neural response magnitudes to object categories at different eccentricity positions.

## Materials and Methods

### Subjects

Seven subjects participated in the study (5 males and 2 females), aged 21–29 years. All subjects had normal vision. The fMRI experiments were performed at the Hospital of Okayama University and were approved by the Ethics Committee of the Hospital of Okayama University.

### Stimulus Presentation

All visual stimuli were generated using Presentation software (Neurobehavioral Systems, Inc.,) and were displayed using a wide-view visual presentation system (Wang et al., 2013a; Wu et al., 2013). In this system, stimuli were presented monocularly (left or right eye) using a hemispheric screen 52 mm in diameter; the curvature radius of this hemisphere was 30 mm. The subjects viewed the stimuli on a hemisphere, with a mean distance of 30 mm between the subjects’ eyes and the screen. The subjects wore contact lenses to focus on the stimulus, and the visual field of stimulus presentation was 120° horizontal × 120° vertical, or 60° of eccentricity.

### Position Experiment

The object position experiments utilized grayscale images of human faces, houses, animals and cars (Figure 1A). As shown in Figure 1B, the images were arrayed in rings with six eccentricity levels. Figure 1C shows sample images of the face ring at 33° eccentricity. The width of the concentric ring was 10° of visual angle. The gap of each concentric ring was 1° of visual angle. One hundred ninety-two unique images were utilized in this experiment. We chose to use a constant image size because the magnification factors for the peripheral visual field are not known for the LOC. If we scaled the stimulus sizes according to the cortical magnification factor that was calculated for V1 in our previous study (Wu et al., 2012) or for LO-1/2 in a previous study (Larsson and Heeger, 2006), the magnifications at the center and periphery would be quite different; the outer stimuli would be very large closer to the fovea.

The object experiment consisted of four runs of a block-design experiment. In each 8 s block, different images from a single category (faces, houses, animals or cars) were shown at a single eccentricity position. To exclude an influence of background, these images were presented with uniform background. With this presentation method, the amount of space occupied by the images in each category differed but were consistent at each eccentricity (faces:houses:animals:cars = 1.3:1.7:1:1). Each image was presented for 0.8 s with a 0.2 s interstimulus interval. Image blocks were interleaved with baseline blocks (grayscale screen with the fixation point) that lasted for 8 s. Each run contained one block for each position and category combination; thus, the session contained 24 blocks per run (4 categories × 6 positions). During the scanning, the subjects were asked to categorize the images while maintaining fixation and to respond by pressing buttons. The fixation disk dimmed randomly at 1.8–3.8 s intervals. The subjects were asked to respond when the fixation disk dimmed. Button presses that occurred outside the 1.2 s period following a response prompt were ignored. During scanning, the behavioral responses were collected using a magnet-compatible button box that was connected to the stimulus computer.

### Localizer Experiment

The localizer experiment was used to define the object-selective area (LOC). The stimuli consisted of 30 grayscale images (22° × 22°) of faces, houses, animals, and cars as well as phase-scrambled images of these images. The experiment was initiated and ended with 12 s of rest and contained 20 stimulus blocks (10 s in duration) that were separated by 10 s blocks of rest. In each stimulus block, 10 images from a single stimulus category were presented, and two or three images were repeated. The subjects were asked to fixate on a central fixation point in the visual field and to respond by pressing a button when an image was repeated.

### Retinotopic Mapping Experiment

Clockwise rotating wedge and expanding ring stimuli were employed to identify the retinotopic areas of the visual cortex (Sereno et al., 1995; Engel et al., 1997; Wu et al., 2012). A red fixation disk (approximately 1°) was presented at the center of the stimuli. These retinotopic stimulus apertures contained high-contrast, black-and-white checkerboard patterns that phase-reversed at a temporal frequency of 8 Hz, with an eccentricity that ranged from 2.4° to 60°. The wedge checkerboards included boundaries of 22.5° and slowly rotated clockwise around the red fixation disk. The wedge rotated at steps of 22.5° and remained at each position for 8 s. These checkerboard rings expanded from 2.4° to 60° eccentricity. These expanding ring stimuli were moved in discrete steps and remained at each position for 8 s. Six cycles of the rotation and expansion of the checkerboard were completed. All experiments employed passive viewing, and the subjects were required to maintain fixation on a red disk throughout the scan period.

### Image Acquisition

Imaging was performed using a 3-Tesla MR scanner (Siemens Allegra, Erlangen, Germany). The functional series included continuously acquired standard T2-weighted echo-planar imaging (EPI) images (TR = 2 s; TE = 35 ms; flip angle = 85°; 64 × 64 matrices; in plane resolution: 2.3 × 2.3 mm; slice thickness: 2 mm, with a gap of 0.3 mm; 30 slices). The slices were manually aligned to be approximately perpendicular to the calcarine sulcus to cover most of the occipital, posterior parietal, and posterior temporal cortices. After the functional scans, one volume of a high-resolution sagittal T1-weighted image (MP-RAGE; TR = 1800 ms; TE = 2.3 ms; matrix 256 × 256 × 224; 1 mm isotropic voxel size) was acquired.

### Data Preprocessing

The anatomical and functional images were analyzed using BrainVoyager QX 2.07 (Brain Innovation, Maastricht, Netherlands). The anatomical images were segmented to identify white/gray matter boundaries and were then used for cortical surface reconstruction and inflating (Goebel et al., 2006). The functional images were preprocessed with scan-time correction, 3D motion correction, and high-pass temporal filtering (0.01 Hz) prior to statistical analysis (Goebel et al., 2006). The functional data were subsequently transformed into the conventional Talairach space, yielding a 4D data representation (Talairach and Tournoux, 1988).

A general linear model (GLM) was applied to the position experiment and localizer experiment data on a voxel-by-voxel basis. A boxcar function was convolved with a double-gamma hemodynamic response function to account for hemodynamic effects (Friston et al., 1998). At the group level, a random effects analysis of variance was performed on the position scans of each subject. A statistical threshold of *p* < 0.05, corrected with the false discovery rate (FDR), and a cluster threshold of 20 mm^3^ were adopted in the statistical analyses. The neural activation maps were rendered on a cortical surface from a high-resolution structural MRI in Talairach coordinates.

### Retinotopic Mapping

The retinotopic maps of polar angle and eccentricity were identified using a linear correlation map analysis. The stimulation blocks were modeled by boxcar functions that were convolved with a double-gamma hemodynamic response function. For each voxel, the stimulus-driven modulation of the BOLD time course was correlated with the response of an ideal response function. This phase was converted into physical units by identifying the stimulus parameter (polar angle or eccentricity) that corresponded to the time. The color-coded cortical regions were classified based on an *r*-value threshold of 0.25. The retinotopic maps were projected onto an inflated cortical surface.

### Region of Interest Analysis

**Figure 2:**
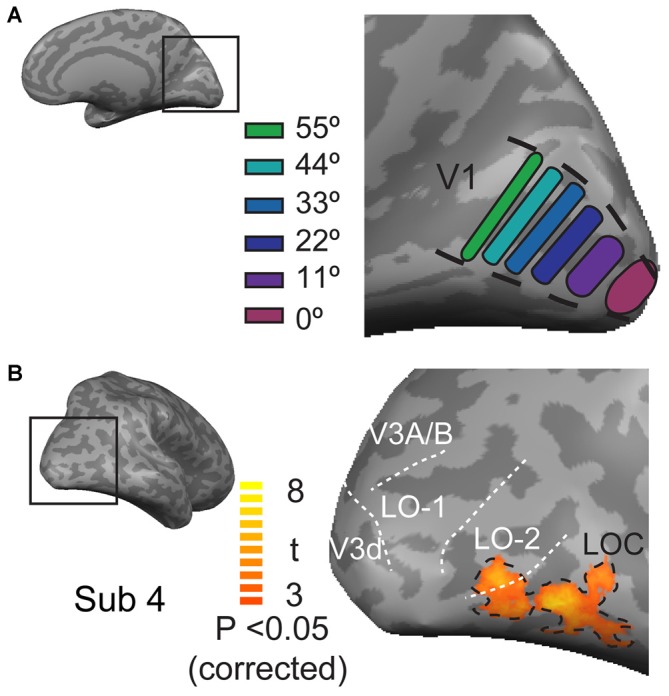
**The locations of ROIs in V1 and the lateral occipital cortex. (A)** The locations of six ROIs at each eccentricity in V1. **(B)** Locations of the LOC, LO-1 and LO-2 in the lateral visual cortex. The black dashed lines outline the position of the LOC, which was defined by contrasting responses to faces, houses, cars, and animals with those to phase-scrambled images. The white doted lines indicate the LO-1, LO-2, V3d, and V3A/B visual areas.

**Figure 3:**
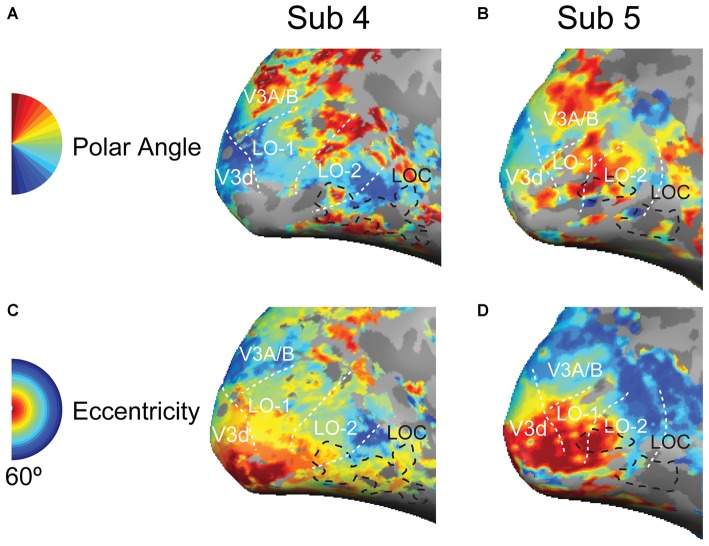
**Retinotopic maps in the lateral visual cortex. (A,B)** Polar angle representations in the lateral visual cortex; the black dashed lines outline the position of the LOC; the white doted lines indicate the LO-1 LO-2, V3d, and V3A/B visual areas. **(C,D)** Eccentricity representations in the lateral visual cortex.

The regions of interest (ROIs) of V1 were individually defined for each participant based on the position experiment data and the V1 mask that was obtained for each individual through retinotopic mapping. This analysis was performed by contrasting the response to the presentation of a stimulus at one position with the responses to the presentation of that stimulus at all of the other positions using a threshold with an FDR-corrected *p* < 0.05. A strip of segments, each with an area of 150 mm^2^, was drawn at the location of neural activation along the calcarine sulcus (V1). In total, six functional ROIs were defined in each hemisphere (Figure 2A). These cortical ROIs were then coveted into 3D volumetric ROIs. The ROIs of the LOC were defined by contrasting all object category images with the scrambled image with a contrast threshold of FDR-corrected *p* < 0.05 and a spatial extent of at least 20 mm^3^ (Figure 2B). LO-1 and LO-2 were identified as reversals in the retinotopic representation, as described by Larsson and Heeger (2006); Figure 3. The neural activation elicited by each object category at each eccentricity position was quantified as the neural response amplitude in each region.

### Relative to the Neural Response in V1

As mentioned above, we did not scale the stimulus sizes according to the cortical magnification factor in V1 and LO-1/2. In addition, all of the images were presented with a uniform background to exclude an influence of the background. Due to this presentation method, the low-level visual properties were unmatched. In the human visual cortex, V1 is considered to be essential for visual information processing. We further scaled the neural responses by the ratio of the neural response relative to that in V1, thereby providing the same input strength to LOC, LO-1, and LO-2 for all eccentricities. We calculated the RRV1 as the neural response amplitude in the LOC, LO-1, or LO-2/the neural response amplitude in V1. When the neural response amplitude in FFA or PPA was greater than that in V1, the RRV1 was greater than 1, and when the amplitude was smaller, the RRV1 was less than 1. Only positive response amplitudes were used for the final calculations.

## Results

### Behavioral Performance

The response time and accuracy of the participants’ recognition of the stimuli as belonging to one of the four categories at each retinal position are listed in Table 1. The constant (no scaling) image size used in the main experiment made it difficult for the participants to categorize the stimuli when they were presented at the far peripheral positions. At eccentricities of 0–33°, the behavioral performance was good; the subjects could recognize image presented in the peripheral visual field but failed to recognize images at the more extreme peripheral positions (eccentricities of 44° and 55°). Some subjects had no or weak responses to the images of faces and houses when they were presented at the most peripheral positions, which resulted in missed responses. Linear mixed models for repeated measures with factors of eccentricity and category (6 × 4) were applied. Accuracy was significantly affected by stimulus eccentricity [*F*_(5,34)_ = 61.3, *p* < 0.001] and category [*F*_(3,44)_ = 27.9, *p* < 0.001]. In addition, there was a significant interaction between category and eccentricity [*F*_(15,23)_ = 7.18, *p* < 0.001], indicating that the discrimination accuracy for each object category was influenced by eccentricity. For example, the accuracy of face image recognition was substantially higher than that for the other image categories at the far peripheral positions (eccentricity of 33–55°), although at an eccentricity of 33°, the accuracy of discriminating houses was less than that for faces and cars. The response time was significantly affected by stimulus eccentricity [*F*_(5,15)_ = 2.6, *p* = 0.01], whereas there was no main effect of category [*F*_(3,29)_ = 2.5, *p* = 0.08]. A pairwise comparison showed that for animal images, response times were shorter for stimuli presented at an eccentricity of 22° than for an eccentricity of 0°.

**Table 1:** **Behavioral results of the position experiment**.

	Category	Eccentricity of Stimulus Position					
		0°	11°	22°	33°	44°	55°
Accuracy(%)	Face	0.84 ± 0.05	0.96 ± 0.02	0.95 ± 0.04	0.98 ± 0.02	0.86 ± 0.04	0.77 ± 0.06
	House	0.86 ± 0.06	0.93 ± 0.04	0.93 ± 0.04	0.64 ± 0.07	0.63 ± 0.12	0.25 ± 0.08
	Animal	0.77 ± 0.07	0.96 ± 0.02	0.84 ± 0.07	0.79 ± 0.1	0.27 ± 0.07	0.14 ± 0.07
	Car	0.79 ± 0.08	0.95 ± 0.03	0.96 ± 0.02	0.82 ± 0.04	0.50 ± 0.09	0.16 ± 0.1

Reaction Time (ms)	Face	684 ± 21	621 ± 28	586 ± 43	580 ± 43	673 ± 38	669 ± 41
	House	725 ± 43	645 ± 39	644 ± 15	715 ± 37	709 ± 48	701 ± 35
	Animal	724 ± 44	643 ± 26	622 ± 41	643 ± 35	680 ± 58	785 ± 25
	Car	762 ± 29	615 ± 33	595 ± 46	651 ± 45	683 ± 49	777 ± 60

*Note: the values are shown as the mean ± SEM*.

### Neural Activation Maps in the Lateral Visual Cortex

**Figure 4:**
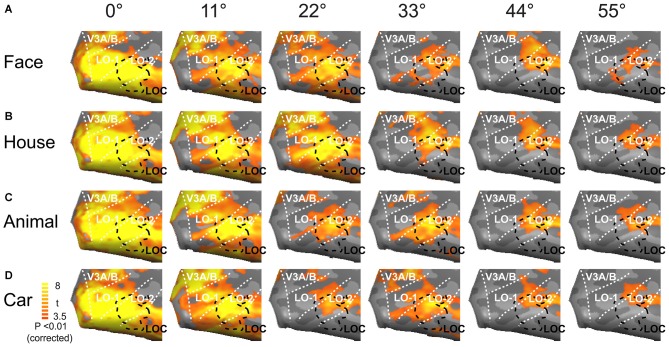
**Mean activation maps in the lateral visual cortex.** Faces **(A)**, houses **(B)**, animals **(C)** and cars **(D)** elicited the strongest neural activations when presented at the central position; the evoked responses became weaker as the images were presented farther away from the visual center, especially at eccentricities of 33, 44 and 55°.

We created neural activation maps in the lateral visual cortex in response to the presentation of objects at six eccentricity levels (Figure 4). The lateral visual cortex (LOC, LO-1, and LO-2) exhibited intense neural activation for each of the six eccentricities, and the maps for four object categories (faces, houses, animals and cars) were similar. As predicted, intense neural activation was elicited by stimuli in the central visual field, and the magnitude of the response monotonically decreased with increasing eccentricity. The activation maps for the six eccentricities substantially overlapped. Objects presented at the central position evoked the strongest neural activation, and these activation maps covered most of the lateral visual cortex. Peripherally presented objects elicited weak neural activation, and these activation maps covered the anterior portion of the lateral visual cortex, which mainly represents the peripheral visual field (Figure 4).

### Neural Response Magnitudes

**Figure 5:**
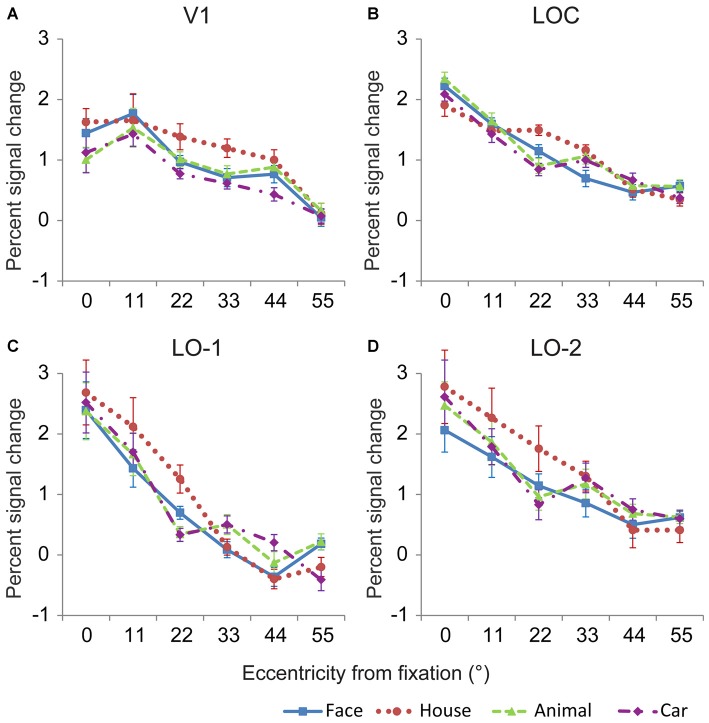
**Mean response amplitude to the four categories in V1 and the lateral visual cortex.** In general, the relationships between eccentricity and the neural responses in V1 **(A)**, the LOC **(B)**, LO-1 **(C)** and LO-2 **(D)** differed significantly, demonstrating that these regions contain eccentricity information. In addition, significant differences in the responses to different categories were identified for most eccentricity positions, indicating that these regions contain category information.

The neural response magnitudes were pooled across both hemispheres, and the averaged response magnitudes are shown in Figure 5. Generally, the neural response magnitudes in the investigated areas progressively decreased from central positions to peripheral positions. Linear mixed models for repeated measures with factors of eccentricity and category (6 × 4) were used to analyze the neural responses in the investigated regions. In V1, there were significant main effects of eccentricity [*F*_(5,94)_ = 54.64, *p* < 0.001] and category [*F*_(3,68)_ = 5.82, *p* = 0.002], and no interaction between eccentricity and category [*F*_(15,61)_ = 1.08, *p* = 0.4] (Figure 5A). The neural responses to house images were larger than those to images of the other categories, possibly due to larger amount of space occupied by the house images. Pairwise comparisons revealed that significant differences between object categories were mainly found at eccentricities of 0, 22, 33 and 44° (*p* < 0.05). In particular, the neural responses to house images were larger than that to animal image at 0° eccentricity, and those to images of other categories at 22 and 33°.

The neural responses in the LOC showed a significant main effect of eccentricity [*F*_(5,83)_ = 158.19, *p* < 0.001] and a significant interaction between eccentricity and category [*F*_(15,53)_ = 4.34, *p* < 0.001] (Figure 5B). Pairwise comparisons revealed that significant differences between object categories were mainly found at eccentricities of 0, 22, 33, and 55° (*p* < 0.05). In particular, the neural responses to face images were larger than those to house image at 0° eccentricity. However, the neural responses to house images were larger than those to face, animal and car images at 22° eccentricity. The neural responses to face images were smaller than those to the other object categories at 33° eccentricity (*p* < 0.05).

The localizer experiment used 22° × 22° images; as a result, the LOC neural responses to localizer stimuli presented at the center of the visual field might be distributed. Therefore, we also measured neural response magnitudes in LO-1 and LO-2, which were defined by their retinotopic representation of the visual field. Responses in LO-1 and LO-2 also differed with respect to eccentricity and category (Figures 5C,D). Using the linear mixed models method, we identified a significant main effect of eccentricity in LO-1 [*F*_(5,70)_ = 51.39, *p* < 0.001] and LO-2 [*F*_(5,82)_ = 31.5, *p* < 0.001]. More importantly, there was an interaction between eccentricity and category [LO-1: *F*_(15,47)_ = 4.36, *p* < 0.001, LO-2: *F*_(15,57)_ = 1.98, *p* = 0.03]. LO-1 and LO-2 exhibited similar category biases at eccentricities of 11 and 33°. House images evoked larger neural responses than the other object categories when presented at an eccentricity of 22°. At 33° eccentricity, face images elicited smaller neural responses than the other object categories in LO-2. In LO-1, responses to faces were smaller than those to animals and cars.

### Relative to the Neural Response in V1

**Figure 6:**
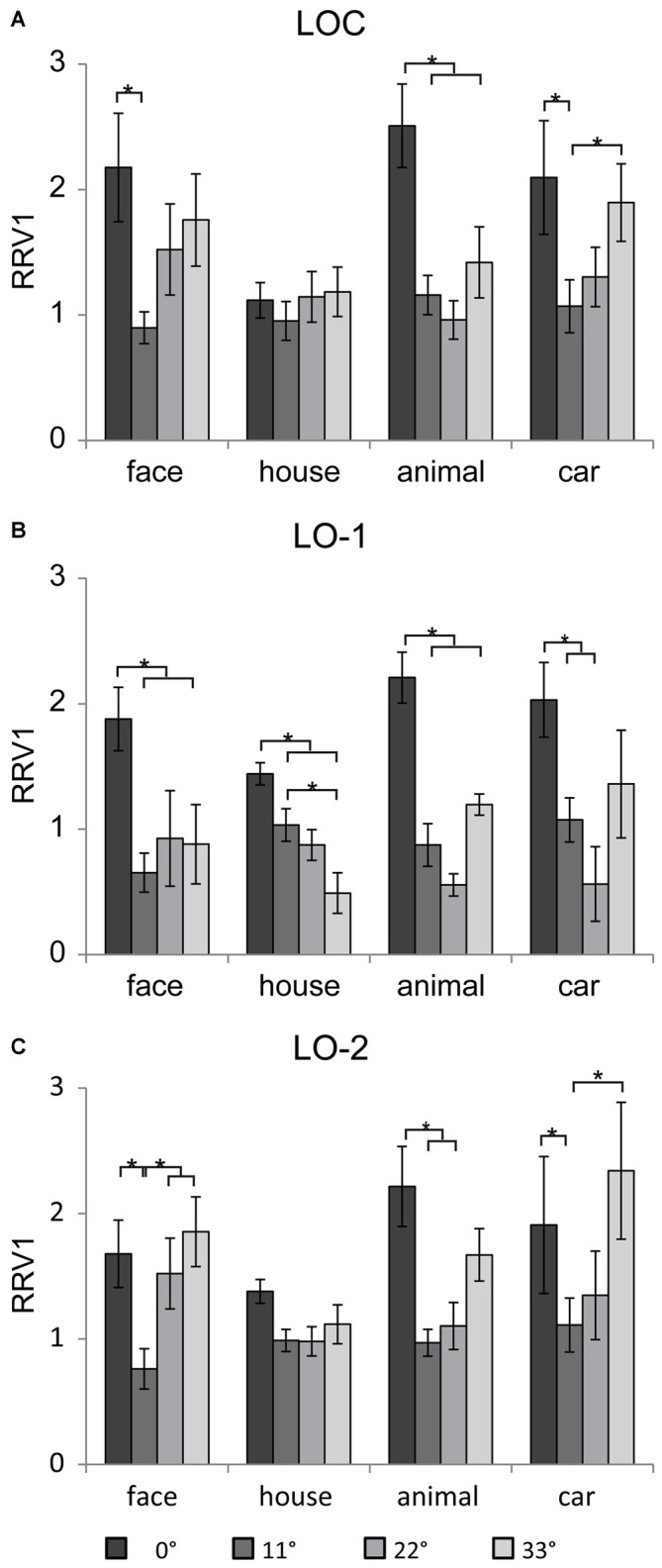
**Mean RRV1 of the four categories in the lateral visual cortex.** In general, the relationships between eccentricity and the neural responses in the LOC **(A)**, LO-1 **(B)** and LO-2 **(C)** differed significantly, demonstrating that these regions contain eccentricity information, which differed for houses and images in the other categories. Significant differences (*p* < 0.05) in individual contrasts are indicated with asterisks.

The neural responses were scaled by the RRV1, ensuring that the strength of the input to the LOC, LO-1, and LO-2 was the same for all eccentricities and categories. In addition, the subjects failed to recognize objects beyond 33° (at the high eccentricities of 44 and 55°), and the neural responses in V1, the LOC, LO-1, and LO-2 were weak. For this reason, we omitted the results for RRV1s beyond 33°. Figure 6 shows the mean RRV1 for each eccentricity for each ROI. Linear mixed models for repeated measures with factors of eccentricity and category (4 × 4) revealed a main effect of eccentricity in the lateral visual cortex (LOC: [*F*_(3,39)_ = 9.4, *p* < 0.001], LO-1: [*F*_(3,56)_ = 17.15, *p* < 0.001], and LO-2 [*F*_(3,69)_ = 13.72, *p* < 0.001]). In addition to eccentricity, there was a significant main effect of category in the LOC [*F*_(3,73)_ = 5.11, *p* = 0.003]. The RRV1s for house images were significantly smaller than those for the other categories (*p* < 0.05) at an eccentricity of 0° and marginally significantly smaller at an eccentricity of 33°. We also identified an interaction between eccentricity and category in the LOC [*F*_(9,45)_ = 2.4, *p* = 0.02] and LO-1 [*F*_(9,48)_ = 3.23, *p* = 0.004]. Pairwise comparisons revealed differences in eccentricity for each category; these differences are indicated with asterisks in Figures 6A,C (*p* < 0.05). In the LOC and LO-2, the RRV1s for face images at an eccentricity of 11° were smaller than those for face images at the other eccentricities (*p* < 0.05). For the images of animals and cars, the RRV1s at eccentricities of 11 and 22° were significantly smaller than those at an eccentricity of 0° (*p* < 0.05). In addition, the RRV1s for car images presented at an eccentricity of 11° were smaller than those for car images presented at an eccentricity of 33°. In LO-1 (Figure 6B), faces, animals, and cars had larger RRV1s when presented at an eccentricity of 0°, while the RRV1s for these stimuli did not differ when they were presented at the outer eccentricities (11–33°). In contrast, the RRV1s for houses presented at eccentricities of 11 and 33°differed (*p* < 0.05).

## Discussion

### Object Discrimination in the Central and Peripheral Visual Field

Evidence from behavior performance and neuroimaging results indicates that the ability of the visual system to discriminate and identify objects decreases as eccentricity increases (Sayres and Grill-Spector, 2008; Yao et al., 2011; Yoo and Chong, 2012). The variance in these abilities is thought to be related to the smaller cortical magnification and larger receptive field size in the peripheral visual cortex; the visual system represents central stimuli with a fair degree of fidelity, but it more crudely encodes stimuli in the peripheral visual field. In the present study, by presenting stimuli in a wide-view field, we also demonstrated that object recognition performance declined when stimuli were presented in the more peripheral visual field. The constant (no scaling) image size used in the peripheral visual field made it difficult for the participants to recognize the category of the presented object. Because the recognition used here was relatively simple, the accuracy rate might be inflated. Subjects were asked to recognize the category of images that were sequentially presented in a single block. For example, if the subject noticed that one image in the block was a car, they would then know that the other images in the block were cars. Thus, we believe the actual ability of subjects to recognize objects in the periphery may be much lower. Our behavior results showed that the subjects could recognize objects that were presented at eccentricities of 33°, but failed to recognize objects at the far peripheral positions (eccentricities of 44 and 55°), similar to several previous reports (Yao et al., 2011; Yoo and Chong, 2012). These findings demonstrate that the ability to recognize objects greatly decreases beyond an eccentricity of 33°.

Moreover, in the peripheral visual field, the discrimination accuracy for each object category differed considerably; at peripheral positions, the accuracy of face recognition was substantially higher than thot for the other categories. Yoo and Chong (2012) also reported higher accuracy for face memory than for house memory. The subjects may have been better able to recognize peripherally presented faces by detecting the first-order relations that define faces and through holistic processing. Holistic processing might be better than part processing in the periphery.

In the peripheral visual field (eccentricity of 33°), the accuracy of face discrimination was higher than that for the other categories. In contrast, at an eccentricity of 33°, the accuracy of house discrimination was smaller than that for faces and cars. In the regions in the lateral visual cortex, we also found that the RRV1s for houses were slight smaller than those for the other object categories at an eccentricity of 33°. Furthermore, it is well known that the face-selective area FFA and PPA are selective for the processing of faces and houses, respectively. The FFA showed higher RRV1s, with a significant increasing trend, while the PPA showed smaller RRV1s and lacked a significant increasing trend (Wang et al., 2013b). It is likely that for presentations in the peripheral visual field, the superior recognition performance for faces was related to the higher RRV1s and the lower recognition performance for houses was related to the smaller RRV1s in the higher visual areas.

### Eccentricity Effect on Object Activation Maps

The activation maps for each eccentricity greatly overlapped, which is unlike maps in V1 (Wang et al., 2013b; Wu et al., 2013). The overlapping activation maps in the lateral visual cortex may be caused by the larger receptive field size, which ranges from 2.8° to 26° (Op De Beeck and Vogels, 2000; Yoshor et al., 2007); similar population receptive field sizes have also been found in human neuroimaging studies (Amano et al., 2009). Within the lateral visual cortex, neurons that represent peripheral visual space exhibit large receptive field; therefore, these neurons respond not only to the peripheral visual field but also to the central visual field. In addition, different numbers of neurons may have been activated by the central and peripheral stimuli. We propose that central presentation activates a large number of neurons whose receptive fields extend from the center to the periphery, whereas peripheral presentation activates only neurons whose receptive fields do not extend to the center.

### Neural Responses to Objects in the Lateral Visual Cortex

In the lateral visual cortex, the neural responses to object categories were also influenced by eccentricity. Centrally presented objects elicited the strongest neural responses, whereas peripherally presented objects evoked substantially weaker neural activations, especially at eccentricities of 44 and 55°. These results are similar to previous results for the central visual field (Sayres and Grill-Spector, 2008) but cover a larger range of the visual field.

LO-1 and LO-2 showed different patterns of eccentricity differences. LO-1 exhibited significantly positive responses only to more central positions (0–22°), whereas LO-2 exhibited significantly positive neural responses to all positions (0–55°). These differences in neural responses appear to be consistent with the differences in eccentricity representations in the visual cortex found in the present study and in previous reports (Larsson and Heeger, 2006; Sayres and Grill-Spector, 2008; Amano et al., 2009). LO-1 represented only the central visual field, whereas LO-2 exhibited a sudden transition from central to peripheral locations.

In the present study, stimulus sizes were not scaled according to the cortical magnification factor in V1; as a result, the stimulus sizes at outer eccentricities were quite large. In addition, the space occupied by houses was larger than that occupied by the images in the other categories; accordingly, the neural responses to house images were larger than those to the images in the other categories. We used RRV1s to provide the same strength of information from V1 for all eccentricities and categories. The RRV1s in the lateral visual cortex showed significant eccentricity effects; this effect differs from the increasing trend found in the FFA and PPA (Wang et al., 2013b). The LOC, LO-1, and LO-2 had larger RRV1s for stimuli presented at an eccentricity of 0° compared to at the outer eccentricities. We hypothesize that different strategies were adopted to process the central and peripheral information from V1. However, low-level visual properties may provide an alternative explanation. In the present experiment, only one image was presented at an eccentricity of 0°, whereas several images were presented at the outer eccentricities. The obvious difference in the number of images might result in the larger RRV1s at an eccentricity of 0°, compare with the outer eccentricities.

The human retina has much weaker visual information processing capabilities in the peripheral visual field than in the central visual field (Curcio et al., 1987; Curcio and Allen, 1990). In a previous report, we found that in the FFA, RRV1s were greater for peripheral positions than for central positions. The larger RRV1s might reflect a compensatory mechanism for the peripheral field in the higher visual cortex. In the present study, similar results were found in the LOC and LO-2. For eccentricities from 11° to 33°, faces, animals and cars showed a trend toward an increasing RRV1 in LOC and LO-2. Based on our findings, we hypothesize that in the LOC and LO-2, compensatory strategies were used to process the information from V1 about the presentation of some of the object categories in the peripheral visual field.

### Category Biases in Neural Responses to Object Categories

In addition to the eccentricity effect, the neural responses to objects and RRV1s in the lateral visual cortex also exhibited category biases. When the images were presented at the central position (eccentricity of 0°), we found that faces evoked larger responses than houses in the LOC but not in V1. In the LOC, we also found lower RRV1s for house image compared to the other categories for presentations at an eccentricity of 0°. Sayres and Grill-Spector (2008) also demonstrated slightly higher responses to animate categories (faces, body parts, and animals) than to inanimate categories (cars, houses, and sculptures) by presenting stimuli only in the central visual field.

At the outer eccentricities (11 to 33°), we found that the neural responses for house images were larger than those for images in the other categories (Figure 5); this difference is likely related to the larger space occupied by the houses and the corresponding larger neural response to houses in V1. Moreover, the space occupied by faces was slight larger than that occupied by the cars and animals. However, at 33° of eccentricity, the smaller response to faces than to cars and animals were found in lateral visual cortex, but not in V1. These findings might reflect the basis of neural activation in peripheral visual field. By analyzing the RRV1s, we identified a significant interaction between eccentricity and category in the LOC and LO-1. The RRV1s for house images were marginally significantly smaller than those for images in the other categories when presented in the peripheral field (Figure 6). More interestingly, as eccentricity increased, the RRV1s for faces, cars and animals showed an increasing trend, whereas the RRV1s for houses showed a decreasing trend in LO-1 and consistent values in the LOC and LO-2. Moreover, in a previous study, we found that in the FFA, RRV1s differed significantly according to eccentricity; in contrast, this relationship was not found in the PPA, suggesting that compensatory mechanisms for the peripheral field may be in the FFA and not in the PPA (Wang et al., 2013b). We further hypothesize that in the high visual areas, different strategies were used to process peripheral information for house images compared with images in the other categories.

Our finding of biases in RRV1 related to object categories might be related to the response accuracy results at an eccentricity of 33°, in that the accuracy for house images was much smaller than that for the other categories. Because the recognition task used here was relatively simple, the accuracy rate might not be sufficient to reflect differences in RRV1 at the inner eccentricities. Our findings are consistent with those of Yoo and Chong (2012), who reported that house memory performance was worse than that for face memory in the central and peripheral visual fields. Thus, the new pattern of eccentricity biases exhibited by the lateral visual cortex might relate to the processing of the category of objects presented in the central and peripheral visual fields.

## Conclusion

In present study, we investigated the neural activation to objects presented in a wide-view field in V1 and the lateral visual cortex, which included the LOC, LO-1, and LO-2. These neural responses in these regions decreased as the distance between the presentation location and the center fixation point increased, but the patterns of neural responses between the regions differed. The LOC and LO-2 exhibited significantly positive neural responses to all eccentricities (0–55°), but LO-1 exhibited significantly positive responses only to central eccentricities (0–22°). Importantly, the magnitude of the neural responses elicited by the different object categories significantly differed. Eccentricity and category, as well as the interaction between them, significantly affected RRV1s in the lateral visual cortex. LOC, LO-1, and LO-2 had larger RRV1s for stimuli presented at an eccentricity of 0° than for those presented at the outer eccentricities. While the house images did not, the images of faces, animals and cars showed a trend toward an increasing RRV1 for eccentricities from 11 to 33°, suggesting that the LOC and LO-2 utilize compensatory strategies for the processing of these images in the peripheral visual field. However, the RRV1s for houses showed a decreasing trend in LO-1 and consistent values in LOC and LO-2. We further hypothesize that the strategies used by the lateral visual cortex to process information from V1 differed with respect to the category of image presented in the peripheral visual field.

## Author Contributions

Conceived and designed the experiments: BW, JG, TY, JW. Performed the experiments: BW, JG, SO, SK. Analyzed the data: BW, JG. Wrote the article: BW, JG, TY, QH, JW.

## Conflict of Interest Statement

The authors declare that the research was conducted in the absence of any commercial or financial relationships that could be construed as a potential conflict of interest.
